# Dr. Hull's Observations on Phlegmatia Dolens

**Published:** 1801-07

**Authors:** John Hull

**Affiliations:** Manchester


### *To the Editors of the Medical and Phyſical Journal.*

GENTLEMEN,

IN an ingenious work, lately printed in this town, entitled, *An Inquiry into the Nature and Cause of that Swelling, in one or both of the lower Extremities, which sometimes happens to Lying-in Women*, Part II. the reſpectable and experienced Author, CHARLES WHITE, Eſq. has inſiſted on a particular affection as conſtituting the pathognomonic ſymptom of the diſeaſe named *Phlegmatia Dolens* in my Eſſay, to which I cannot aſſent; has objected to ſeveral caſes, related in my Eſſay, in which all the ſymptoms, that I deem eſſential or characteriſtic, were preſent; has given a theory of the diſeaſe, which I conceive to be purely hypothetical; and has urged various objections, which I think may eaſily be invalidated, to the theory advanced by me. I am therefore induced to lay before the public ſome remarks upon Mr. White's publication, and will thank you to give them a place in your valuable Journal for next month. I have the honour to be, &c.

*Mancheſter, June* 12, 1801. JOHN HULL.

---

THE OBSERVATIONS which I have to offer upon Mr. White's *Inquiry*, relate principally to the diagnoſis of the diſeaſe, which he has choſen to name *Phlegmatia alba dolens puerperarum*; to the theory of this diſeaſe, given by Mr. White; and to the arguments urged by him againſt the theory which I have endeavoured to eſtabliſh.

I. *Observations on the Diagnosis of Phlegmatia Dolens.*

"The PATHAGNOMONIC SYMPTOM of this diſeaſe," ſays Mr. White, "is a ſwelling of the whole labium pudendi on the ſame ſide *only*, on which there is a firm, gloſſy, warm, tenſe, elaſtic, painful, ſudden ſwelling, of a pale white colour, which attacks the hypogaſtric region, the loins, nates, groin, thigh, leg and foot of a lying-in woman; and I muſt beg leave to impreſs this upon my readers, that when one limb only is affected, the ſwelling is confined ſo exactly to the labium pudendi of that ſide, that if a line were drawn from the navel to the anus, it would be found never to go beyond that line in the ſmalleſt degree." *Inquiry*, &c. Part II. p. 7.

Admitting, for the preſent, that the labium pudendi is invariably affected in the manner ſtated above, when the diſeaſe affects only one of the lower extremities, this affection is, I apprehend, very improperly ſtyled the pathognomonic ſymptom of Phlegmatia *dolens*. It includes the whole concourſe or aſſemblage of ſymptoms, by which we judge of the preſence of the complaint in that individual part. The ſame ſymptoms, occurring in the nates, inguen, or hypogaſtrium, conſtitute the diſeaſe in theſe reſpective parts; taking place in the thigh or leg, they indicate the preſence of the diſeaſe in theſe parts alſo. And no ſolid reaſon can be aſſigned, why pain, heat, tenderneſs, and ſwelling of the labium pudendi, unaccompanied by redneſs, ſhould be taken as the pathognomonic ſymptom by the Author of the *Inquiry*, &c. in preference to the affection of the nates, thigh, inguen, or hypogaſtrium, ſince each of theſe parts is at leaſt as frequently affected as the labium pudendi, in the complaint under conſideration. The ſwelling of the labium pudendi, in my opinion, is to be conſidered only as *marking the extent*, not as *serving to distinguish the disease*: Whence I contend that Phlegmatia *dolens* may be as certainly diſtinguiſhed, without making a ſingle inquiry, relative to the ſtate of the pudendum of the patient, as by the moſt curious and ſcrupulous examination of this part of the body. This, however, would not be the caſe, if the affection of the labium were the pathognomonic ſymptom, or an eſſential part of the diſeaſe. Acute pain of the thorax in Peripneumonia *Pleuritis*; of the abdomen in Enteritis *phlegmonodea*, are truly pathognomonic ſymptoms, and no practitioner can determine upon the preſence of theſe diſeaſes, without knowing the pain to be preſent in them.

That the labium pudendi of the correſponding ſide, and of this ſide only, is affected with ſwelling, pain, &c. &c. in every caſe of this diſeaſe, I cannot, however, grant. This affection did did not occur in the cafe of Maffey, which I fhall have occafion to mention more particularly hereafter, on either fide, although both her lower extremities were affected in fucceffion, as well as the inguinal and hypogaftric regions. It occurred on both fides in the cafe of Mrs. ********, to be mentioned hereafter, although only the left lower limb was affected with the complaint under difcuffi n. Mr. Tomlinfon informs me, that he has known the fwelling of the labium pudendi wanting in this difeafe; and Mr. Wood has alfo made me acquainted with a recent cafe of this complaint, which he confiders as a well marked one, in which the pain and fwelling began in the leg, and the labium pudendi remained unaffected. Of this I muft beg leave to fubjoin a fhort account: Mrs. D***, of Salford, was delivered on November 17, 1800. On the 24th, fhe had pain in her back, and pain and enlargement in one of her breafts. The day following fhe experienced fome pain in her head, was thirfty, had a frequent pulfe, and other febrile fymptoms. On December 1ft, fhe had rigors fucceeded by heat, pain in the head, thirft, frequent pulfe, and was alfo attacked with a pain in the calf of her right leg. This pain, which was very violent, extended at firft upwards to the knee, thigh, groin, and hypogaftrium, and was followed by a fwelling of thefe parts, it alfo extended down the leg to the foot. The whole limb became much fwelled, tenfe, and tender, was not at all difcoloured, and fo firm and elaftic as not to pit on preffure. On the fourth day of the complaint a lymphatic gland in the groin became enlarged to about the fize of a walnut; but there was no perceptible enlargement or inflammation of any abforbent veffel or veffels. The other leg was not affected with the complaint. If this be not a genuine cafe of Phlegmatia *dolens*, I fhould wifh to know what other difeafe it can be referred to?

If Mr. White really confiders the affection of the correfponding labium pudendi as an indifpenfable part, a *sine qua non*, of the difeafe, how has it happened that *he* could recognize the difeafe in the defcriptions, hiftories, and characters of it, given by the different practical and nofological writers who preceded him? Puzos, to whom we are indebted for the firft clear and accurate account of Ph. *dolens*, Levret, Sauvages, Sagar, &c. are totally filent as to any affection of this part.

That Mr. White himfelf muft confider the complaint as diftinguifhable, independently of what he has been pleafed to ftyle the *pathognomonic symptom*, appears evident from his own Preface, page 8, where he fays, "The complaint had not indeed wholly efcaped his countryman Sydenham, for though he ranked it in a different clafs of difeafes, viz. Hyfterical, yet under der that head *he has described* it with other difeafes of child-bed women." In proof of this affertion he gives the following extract from Sydenham's *Diss. Epistol. ad G. Cole:* "In quo genere is, *qui tibias distendit*, tumor præ reliquis confpicuus eft. Cum enim in Hydropicorum tumoribus, duo hæc femper obfervari poffint, ut tumor fcilicet fub vefperam fe majorem exhibeat, tum etiam ut, paftæ inftar madentis, veftigium digiti fortius impreffi, formamque, referat; *jam in hoc altero tumor matutino tempore protuberantior est, nec digito cedit prementis, aut vestigium ullum manet.* Plerumque etiam tibiarum alterutram tantum inflat: de cætero five magnitudinem fpectes, five etiam fuperficiem, Hydropicorum tumores ita ad amuffim æmulatur, ut ægræ fe haud facile ab ea, quæ infedit fententia, dimoveri patiantur." Yet in this extract, Sydenham does not mention any fwelling of the labium pudendi, or groin, or hypogaftrium, or even the thigh; he notices an *elastic* fwelling of the leg, but he does not even fay that this was attended with pain, forenefs, or heat; fymptoms which other writers, as well as myfelf, deem effential to, or characteriftic of, Ph. *dolens.* Hence, if the affection, defcribed by Sydenham, be referrable to the genus Phlegmatia, it would appear to belong to the fpecies that I have named Ph. *diuturna*, rather than to Ph. *dolens.* But even this can hardly be allowed. We have the high authorities of three nofologifts, Sauvages, Cullen, and Sagar, for referring the difeafe mentioned by Sydenham to the genus Pneumatofis, each of whom have named it Pneumatofis *hysterica.* See *Sauv. Nos. Meth. T.* II. *p.* 273, (where it is defcribed in Sydenham's own words); *Sagar Syst. Morb. Sympt. p.* 150; *Cullen Syn. Nos. Meth. Vol.* II. *p.* 150—Had Mr. White attended carefully to the four or five lines immediately preceding the above quotation, he would have perceived that Sydenham was treating of a very different complaint: "Atque ut hic morbus," fays this celebrated writer, fpeaking of *Hysterical Affections*, "omnes fermè partes *internas* infeftat, ita etiam nonnumquam et *exteriores* carnemque mufculofam occupat, *maxillas* fcilicet, *humeros*, *manus*, *crura*, *tibias*, nunc dolorem adducens, nunc tumorem." *Opera Universa.* P. 391, 8vo. Ludg. Bat. 1741.

As far as I have been able to difcover, Mr. White is the firft writer who has taken notice of the fwelling, &c. of the pudendum in Phlegmatia *dolens.* Since his publication appeared, it has been noticed by Dr. Evans, Callifen, and me, (*Essay*, p. 134); but neither Callifen nor I have mentioned it as an effential circumftance.

Mr. White coincides with me in opinion, that this complaint is admirably defcribed in the *Princ. Syst. Chir. Hod.* of Callifen; let us therefore attend to what is there faid upon the fubject.

fubject. He begins with giving the following definition or character of the difeafe: " Œdema puerperarum, aliis lacteum, eft tumor elafticus, albefcens, renitens, calidus, dolens, foveam, impreffi digiti haud retinens, puerperis haud infrequenter, gravidis rariffime infeftus." Part II. § 34. Now, a *definition* or *character* of a difeafe confifts of the effential fymptoms, and is thereby contra-diftinguifhed from a *description* or *general history*, which, befides thefe, comprehends other fymptoms that are not effential, or all that occur in the courfe of this difeafe. By omitting to mention the labium pudendi in this paragraph, Callifen has fhewn that he does not regard the fwelling of this part as an effential or neceffary circumftance. The effential or characteriftic fymptoms, according to this excellent writer, are an elaftic, white, firm, hot, painful tumour, that does not retain the impreffion of a finger, and which occurs not uncommonly in lying-in women, but very rarely in pregnant women. Whoever will take the trouble to read the generic character of Phlegmatia and the fpecific character of Phlegmatia *dolens*, as given in my Effay, (page 354) will find that I have not differed materially from Callifen. So far indeed is this author from confidering the affection of the labium pudendi as a neceffary part of the difeafe, that he informs us the complaint, though very rarely, *affects the upper extremities*, " Rariffime extremitates fuperiores petit." § 35.

In the defcription of the difeafe, as affecting one of the lower extremities, Callifen takes notice of its affecting the vulva; but inftead of agreeing with Mr. White, that " the fwelling is confined fo exactly to the labium pudendi of that fide, that if a line were drawn from the navel to the anus, it would be found never to go beyond that line in the fmalleft degree," he fays it affects both the labia: " Surgit plerumque 12mo. vel 14to. poft partum die tenfio et dolor inguinis, fequitur tumor, qui fenfim ad *labia* vulvæ et latus femoris internum, deinde per totum femur extenditur." § 36. And afterwards, in laying down the marks by which this complaint may be diftinguifhed from *Oedema hydropicum*, he does not once allude to the affection of the vulva.

Mr. White appears to me to have taken the fame individual cafe both as a cafe and as not a cafe of Phlegmatia *dolens*. The cafe to which I am alluding, is one related by Dr. Evans, in the 10th vol. of the Firft Decade of Duncan's Med. Comm. p. 302—304, and copied by Mr. White in his *Inquiry*, P. II. p. 18—20. Since there is an error in one of the dates, as given by Mr. W. which rendered the hiftory of the difeafe unintelligible to me, I fhall fubjoin a fhort account of the cafe with Dr. Evans's remarks upon it.

" A lady

"A lady of a delicate conftitution, being about 3 months advanced in her pregnancy, was (on the 19th of June, 1783) fuddenly alarmed by an uterine hæmorrhage." On the next day, as the difcharge continued in fmall quantity with flight pain in the loins, ten ounces of blood were taken from the arm. A draught compofed of the Dec. cort. per. rubr. with ac. vitr. was given every four hours, and an anodyne at bed time. This treatment, with the addition of a bolus of nitre and camphor, which was taken with each draught, was continued for ten days. On the 30th, fhe complained of a chillnefs over the whole body. On the 1ft of July, in the morning, "fhe had a pain in the left groin, *a swelling of the labium pudendi of the same side*, which extended to the back, accompanied with a dyfuria, a pain and fwelling down the infide of the thigh and the back part of the leg, with fuch a degree of ftiffnefs as to prevent the extenfion of the limb without great uneafinefs." "On the *ninth* of July, the fwelling of the whole limb was increafed and had an œdematous appearance, but was harder to the touch than in a common anafarca." "On the 27th, in the afternoon, the fwellings fuddenly difappeared," and the next morning fhe mifcarried. "Every fymptom of difeafe now difappeared, and nothing remained but a fmall degree of fwelling towards night, which gradually diminifhed as fhe gained ftrength." "It is evident," Doctor Evans fays, "from the cafe here related, that the difeafe is not peculiar to women in the ftate defcribed by Mr. White, therefore the caufe of this complaint is ftill obfcure, and is likely to remain fo, till diffection affords an opportunity of inveftigation." We have here the pathognomonic fymptom fo much infifted upon by Mr. W. and yet it feems that he does not admit this to be a genuine cafe; for he fays, "Though I am decidedly of opinion, that the *Phlegmatia alba dolens puerperarum* can never be occafioned by preffure, yet I can conceive, *that a temporary complaint fimilar to it*, may be brought on in the early months of pregnancy, (viz. about the third or fourth) by the impregnated uterus preffing the lymphatics againft one fide of the pelvis." *Inquiry*, *&c.* P. II. p. 18. And he then relates the above cafe as an inftance. He afterwards clearly admits, that this was a cafe of the difeafe, and oppofes it to the theory which I maintain; fince he fays, "The cafe related by Dr. Evans is a complete refutation of the doctrine of inflammation of any kind being the caufe of this complaint, *as the lady was of a delicate conftitution, and had not one inflammatory fymptom*, but had an uterine hæmorrhage, and had ten ounces of blood taken from her arm." *Ibid.* p. 21. I admit this cafe as a true and genuine cafe of Ph. *dolens* occurring during pregnancy, but, but I cannot accede to the inference from it made by Mr. White. Delicacy of conftitution does not neceffarily exempt a lady from inflammatory complaints; and it is not expreflly ftated, whether there were any inflammatory fymptoms or not: but if the uterine hæmorrhage were not believed to be of the active kind, I afk, Why was this delicate lady bled from the arm to the amount of ten ounces? I fhould infer from this treatment, that there was an inflammatory diathefis. And it appears highly probable to me, that after the bark had been taken for fome time, the inflammatory diathefis re-appeared, or was increafed; that then the pyrexia fupervened, and in confequence of it, a ftrong determination of the circulating fluids to the limb affected, and effufion of coagulable lymph took place; whence pain, fwelling, and the other fymptoms of Phlegmatia *dolens* were produced. This cafe I cannot help confidering as favourable to the theory I defend, and as completely fubverfive of the theories which fuppofe the difeafe to be occafioned by a divifion or preffure of the lymphatics paffing along the fide of the pelvis. How could the lymphatics be divided, or compreffed here by the preffure of the uterus againft the fide of the pelvis, when the uterus was much too fmall to fill the tubular part of the pelvis? But of this more will be faid hereafter.

Before I proceed to demonftrate, that the objections made by Mr. White to feveral of my own cafes of Phlegmatia *dolens*, are not well founded, I muft beg leave to ftate, that in the character and defcription of this malady, given in my Effay, (p. 354 and 356) the *intumescentia albida*, or *pallida*, is made an effential fymptom; and that in the general hiftory I have expreflly ftated the tumefied extremity to be "*of the natural colour, or even whiter, &c.*" See Effay, p. 136. If therefore, in a fingle cafe, I have omitted to ftate this circumftance particularly, I fhould hope that the reader would confider it as underftood. Prepofterous, indeed, would it be in me, to relate, as genuine hiftories of a difeafe, cafes, in which the fymptoms that I deem effential, did not exift. In the general hiftory of this difeafe, given in the former part of the *Inquiry, &c.* by Mr. White, he fays, "the limb *is not attended with inflammation*;" (p. 8) and afterwards (p. 9) the fwelling "is very fmooth, fhining, and pale." This is the whole he has faid with refpect to the colour of the affected limb in that publication; and in twelve out of the fourteen cafes there related, he makes not the leaft mention of the colour of the limb. In the ninth cafe he fays, "the fwelling of the extremity increafed for fome days, till it was very tenfe, fhining, and *marbled, but without inflammation*," (p. 24); and in the tenth he fays,

"that the parts affected became *pale.*" A perſon, therefore, who infers with Mr. White the non-exiſtence of a ſymptom in a caſe, becauſe it is not expreſſly ſtated, might, on this ground, reject twelve out of the fourteen caſes related by this writer, which, I conceive, would be very unfair, as he has mentioned the paleneſs of the limb in his general hiſtory, prefixed to the particular ones. In the tenth and eleventh caſes, Mr. White has not expreſſly ſtated the affection of the labium pudendi to have been preſent; but it would not be fair in any perſon who may conſider this as an eſſential part of the diſeaſe, to reject them on this account, for he has not ſtated that this ſwelling was wanting. Nine of the women who were the ſubjects of theſe fourteen caſes, are expreſſly ſtated to have been delivered and attended by other perſons; and of the remaining five, I ſhould not ſuppoſe that all were attended by Mr. White; it might, therefore, be objected to the majority of theſe caſes, that he could not have had an opportunity of aſcertaining whether the ſwelling of the labium pudendi extended or not beyond a line drawn from the navel to the anus. But how unfair it would be, to raiſe objections to, and reject Mr. White's caſes, on ſuch grounds as theſe, when it is evident, that he is acquainted with the ſymptoms of the diſeaſe, and it is to be ſuppoſed, that he would not give as particular hiſtories of the diſeaſe, caſes in which theſe ſymptoms were wanting.

I have not objected to any of Mr. White's caſes upon any ſuch grounds as have juſt been ſtated, or indeed upon any ground, in my Eſſay on Phlegmatia *dolens*, nor do I mean to object to any of them in this paper: but I ſhall now ſhew, that Mr. White, in raiſing objections to four caſes which were minutely attended to by me, has not only concluded certain ſymptoms to be wanting, becauſe I have omitted to ſtate them expreſſly, but has alſo ſtated an *eſſential* ſymptom to have been wanting in more than one caſe, which is particularly expreſſed; and this he has done without further inquiry, from reading the caſes, as related in my Eſſay. He has alſo ſelected extracts from my caſes, in order to prove them not genuine, in which is given what I do not deem eſſential to the diſeaſe, and he has omitted to give thoſe paſſages, in which the ſymptoms are particularly mentioned, which I do conceive to be eſſential to, and characteriſtic of the complaint under diſcuſſion. I ſhall now enter upon the proof of what I have juſt advanced.

Speaking of my fourteenth caſe, (the caſe of Sarah Knight) Mr. White ſays, "it wanted two principal ſymptoms, the ſwelling of the *labium pudendi*, and *the white leg.*" *Inquiry*, P. II.

P. II. p. 47. In the relation of this cafe, I have omitted to mention, whether the labium pudendi was affected or not; but I have this day (June 9) learnt from the patient, that the labium pudendi of the correfponding fide only, was feverely pained and tumefied. I have alfo omitted to ftate the colour of the limb, but have obferved, that the pain which began in her right hip, extended to her groin, thigh, and leg, and was "afterwards fucceeded by a tenfe, elaftic, lucid, hot, tender, painful fwelling of the whole limb." *Effay*, p. 175. And Mr. Boutflower, and Mr. C. Boutflower, as well as myfelf, can teftify as to the whitenefs of the limb in this woman.

Mrs. Hulfe, the fubject of my thirteenth cafe, was attended by Mr. Wood and me. Mr White, fpeaking of this cafe, fays, "There are fome circumftances wanting in this cafe to enable me to decide pofitively, whether this was the *Phlegmatia alba dolens*, fuch as the fwelling of the correfponding labium pudendi, and the white leg." *Inquiry*, &c. P. II. p. 45. Neither Mr. Wood nor I attended to the ftate of the pudendum in this cafe, we are confequently unable to fay, either that the fwelling of the labium pudendi was prefent, or wanting in this patient; and as I cannot learn where fhe refides at this time, I cannot make the inquiry: But furely Mr. White is not juftified in faying, that this affection was wanting. With refpect to the whitenefs of the limb, both Mr. Wood and I know that this fymptom was prefent, and the following quotation from my account of the cafe of Mrs. Hulfe will, I truft, convince the reader, that this circumftance has been fufficiently pointed out. "She was feized with a fevere pain in the groin, and fuperior part of the right thigh, which defcended to the leg and foot, and a tenfe, *colourlefs*, elaftic fwelling of the whole limb fupervened." *Effay*, p. 170.

To the cafe of Mrs. ********, which is the 12th in my effay, Mr. White alfo objects, and fays, "the fkin of the limb had not the milky whitenefs, but was inflamed, nor was the fwelling confined to the labium pudendi of that fide, which was affected, but extended to both labia pudendi." Mr. W. then gives what he is pleafed to call my own ftatement of this cafe, and concludes in the following words: "This complaint therefore, decidedly, was not the phlegmatia dolens, but a return of the anafarca which had preceded delivery." *Inquiry*, P. II. p. 45. The cafe of this lady is given at great length in my publication, but as this may not be read by many perfons into whofe hands Mr. White's Inquiry will come, I fhall ftate the following circumftances. Her limbs had been very anafarcous during pregnancy, but the fwelling of thefe had entirely difappeared on the 6th of July, fifteen days after her delivery.

 At

At feven in the evening of this day "I faw her again. She had experienced fome degree of chillinefs foon after I vifited her in the morning, which was fucceeded by heat, and about three P. M. fhe was attacked with violent pain in the left leg. The pain began upon the fore part of the leg, a little below the middle, and extended upwards to the calf. The leg alfo felt hotter than natural, and ftiff when moved. It was foon after examined carefully by the nurfe, who obferved a blufh of fmall extent on the fore part of the leg, *which went off after rubbing it a little.* In half an hour the pain and ftiffnefs affected the whole limb, and were foon followed by a tenfe, elaftic, hot intumefcence, without any difcolouration of the fkin. When I examined the limb, four hours after the attack, *the swelling appeared uniform, extending from the foot to the groin, hip, and lower part of the abdomen. It was hotter than natural, and not at all discoloured, tense, tender, and so firm, that it scarcely retained any degree of impression from the finger,* &c." Effay, p. 154, 155. Upon thefe appearances I form my opinion of this affection being a genuine cafe of Phlegmatia *dolens*. Let us now fee, how much of the above defcription of it is quoted by Mr. White. He only gives thefe words: "July 6th, fhe was attacked with violent pain in the left leg; a blufh of fmall extent on the fore part of the leg." *Inquiry*, p. 43. I afk if this can be called my own ftatement with any degree of propriety? He then adds, "July 27th, both of the labia pudendi were become more enlarged, more tenfe and tender, &c." and afterwards dwells upon the fymptoms, which mark the œdematofe ftate of the limb, fucceeding to Phlegmatia *dolens*. I flatter myfelf, enough has been adduced to fhew, that this affection of the left leg and thigh was truly a cafe of the difeafe to which I have referred it. It forms a ftriking contraft to the œdematofe affection, which appeared in the right leg, after the patient was able to fit up, and which may be ftyled anafarca *puerperarum*.

Refpecting the cafe of Ann Maffey, Mr. White fays, "After delivery there might be thoracic inflammation, but I think it not improbable that it might be hydrothorax. My reafon for being of this opinion is, that fhe had anafarca gravidarum." *Inquiry*, P. II. p. 148. Speaking of the affection of the limbs, which fucceeded, he adds, "that it was not the phlegmatia alba dolens, I am perfectly fatisfied. It not only wanted the pathognomonic fymptom, *the swelling of the corresponding labium pudendi*, and alfo the *tumor albefcens*, but it had fymptoms which are not compatible with this diforder." *Inquiry*, &c. p. 49. Whoever will read the whole account of this cafe, as given in my Effay (p. 178, 186), with attention, will, I think, differ totally totally in opinion from Mr. White. An œdematofe affection occurring in the latter months of pregnancy does not prevent the woman from having an internal inflammation during her lying-in. And the thoracic affection ftands characterized by inflammatory fymptoms, and not by the fymptoms of hydrothorax. This patient had coldnefs and fhivering, fucceeded by heat, head-ach, and other febrile fymptoms. She had pain in the left fide of the cheft, difficult refpiration, and cough. The craffamentum of her blood had a thick buffy coat upon it, and prefented a cupped or concave furface. The fymptoms were fo urgent, that Mr. Stewart, under whofe care fhe was, bled her four times from the arm, applied leeches twice to her fide, and bliftered her twice in the courfe of ten days. I fhall now give fome extracts from this cafe, which fhew the affection of the lower limbs to have been properly confidered as a true cafe of Phlegmatia *dolens*. On the 22d of July, "a pain fuddenly invaded the whole of the left lower extremity; the limb was rubbed, and foon after began to fwell; the febrile fymptoms were aggravated. July 23, *the limb was very painful and much swelled; the swelling was tense, tender, and free from discolouration; it scarcely retained the impression of the finger for any sensible time in the thigh.*" Effay, p. 180. On the 10th of Auguft, when the fwelling of the left lower limb was gradually decreafing, a pain, which fhe had felt for fome time in the back part of the calf of her right leg, "*ascended to her right thigh, groin, and hypogastrium on the fame side, reaching nearly as high as the navel. The inside of the thigh was more particularly painful. The pain afterwards extended to the leg, and the whole limb became swelled, tender, tense, and more or less elastic in different points, but was not discoloured.*" Effay, p. 181. On the 17th of Auguft I faw this woman with Mr. Stewart. "*At that time the right thigh was more swelled in proportion than the leg. It was also more tense and very tender, when touched, especially along the inside. It did not retain the impression of a finger.*" Effay, p. 182. When I attended this patient, I made no inquiry as to the prefence or abfence of fwelling of the pudendum; but I have learnt within thefe few days, that neither of the labia pudendi were fwelled, though the inguinal and hypogaftric regions were confiderably tumefied. I do not acknowledge the incompatibility of any fymptom, or fymptoms, with this malady, that are not contradictory of the fymptoms which I deem effential to and characteriftic of the complaint. It certainly is not an incompatible circumftance, that the cutaneous veins were found enlarged and blue at the top of the thigh of this woman feven days after the attack of the complaint.

Mr.

Mr. White afterwards fays, "there is but one cafe more in Dr. Hull's Effay, which I fhall have occafion to notice, and this will be neceffary, *because it is the only one*, befides Profeffor Zinn's, *where the disease has been supposed to prove fatal.* This cafe of Mrs. C——, of Manchefter, was communicated to Dr. Hull by Mr. Tomlinfon." *Inquiry*, P. II. p. 5[illegible]. In the [illegible]6th page of my Effay, I have given an extract from the works of Puzos, in which he declares, that he has had the misfortune to lofe more than one patient of this difeafe. "J'ai eu le malheur de perdre plus d'une malade, &c." The very firft cafe of Phlegmatia *dolens*, related in my publication, had a fatal termination. The fubject of it was the daughter-in-law of M. Clèments, the mafter of Puzos. And the 5th cafe alfo was a fatal one. The complaint occurred to a lady in the fourth month of her pregnancy in one limb. It then paffed to the other lower limb. She was delivered at the term of feven and a half months. The complaints continued after her delivery, and fhe died on the ninth day. Mr. White, after giving a long quotation from the cafe of Mrs. C——, proceeds to fay: "It does not appear that there was any fwelling of the correfponding labium pudendi, the leg not white, not that milky whitenefs defcribed by Levret, not the *tumour albescens* of Callifen. The fwelling did not begin in the groin or thigh, as defcribed by the beft authors, but in the leg. I muft therefore reject this cafe, as not being the phlegmatia alba dolens puerperarum, but *must attribute her death to a return of the anasarca*, which had preceded her delivery, efpecially fince after her death the leg burft and difcharged a liquid flightly tinged with blood; this being a circumftance which frequently happens in anafarca, but *never* in phlegmatia alba dolens puerperarum." *Inquiry*, P. II. p. 52. Mr. Tomlinfon informs me, that he never inquired into the ftate of the labium pudendi in this lady: But it will appear from the two following extracts from this cafe, as related by Mr. T. that the *tumour albescens* was not wanting: "Her leg was now very much fwelled, and *exhibited the same marks that are usually observed in the painful intumescence of the lower limbs, incident to lying-in women.*" "The whole limb, on examination, was tenfe, confiderably fwelled, and very tender to the touch, *but not discoloured.*" Effay, p. 193. We have the authority of Puzos for faying, that this difeafe has proved fatal; and when this happens, we may, I think, expect the limb in fome inftances to burft after death, and to difcharge a *small quantity* of liquid, *without reducing the limb materially*, as in the cafe of Mrs. C——. On the other hand, where an anafarcous limb burfts after death, we may expect the difcharge of liquid to be be confiderable enough to reduce the fwelling of the limb materially.

Mr. White fays, " I am well fatisfied, that there is not one cafe of the genuine difeafe, in its fimple, uncomplicated ftate, fufficiently authenticated, that ever proved fatal; not one that was ever attended with any external inflammation, abfcefs, gangrene, or burfting of the fkin in the legs or thighs as in anafarca; that though the *pain* fometimes begins in the ham or the calf of the leg, yet the *swelling* never begins fo low." *Inquiry*, p. 8. He adds, " that it never attacks the fame fide more than once." It is painful to me to differ in opinion upon many of thefe and fome other points, relative to Phlegmatia *dolens*, with a profeffional gentleman who has had fo much experience, and juftly acquired fo much celebrity as Mr. White: But I think I have eftablifhed the contrary by incontrovertible facts in my Effay.

I fhall conclude this part of my paper, with obferving, that Mr. White appears to me to have taken a too confined view of the difeafe; to have defcribed a particular variety inftead of a fpecies; and to have improperly excluded all thofe cafes, wherein the difeafe has begun, proceeded, or terminated differently from the cafes which have fallen under his own obfervation, and thofe wherein the affection of the labium pudendi and the whitenefs of the leg have not been particularized. Had fome of his own cafes occurred to a lefs curious obferver, or to a practitioner, who did not conceive the fwelling of the labium pudendi to be an effential part of the complaint, it is very probable that in thefe cafes the tumour of this part would not have been ftated, and that thefe very cafes might have been ranked by Mr. White amongft the exceptionable ones.

( To be concluded in our next Number. )

